# Improving Parent and Therapist Experiences of Codelivering Intensive Upper Limb Interventions for Children With Hemiplegia: A Qualitative Study Using the Theoretical Domains Framework

**DOI:** 10.1111/cch.70040

**Published:** 2025-01-28

**Authors:** Jill Massey, Vicki Tsianakas, Anne Gordon, Glenn Robert

**Affiliations:** ^1^ Evelina London Children's Hospital Guy's and St Thomas' NHS Foundation Trust London UK; ^2^ Florence Nightingale School of Nursing Midwifery and Palliative Care London UK

**Keywords:** cerebral palsy, parent participation, family‐centred service, paediatrics, qualitative research methods, rehabilitation

## Abstract

**Background:**

Partnership working between parents and therapists is a key component of family‐centred care (FCC). Such partnerships in paediatric intervention delivery can help achieve required levels of dosage, intensity and embed interventions in the child's everyday activities. This study explores the experience and views of parents and therapists codelivering an intensive upper limb intervention programme for children with hemiplegia, to find ways to enhance successful partnership working.

**Methods:**

Semistructured interviews were conducted with 12 parents and 8 therapists (3 hospital‐based and 5 community‐based). To help make evidence‐based recommendations, data were analysed using inductive reflexive analysis and mapped to the Theoretical Domains Framework (TDF) of constructs that are common determinants of clinical behaviours or practices.

**Results:**

Five major themes were identified as follows: (1) realities of accessing intensive intervention, (2) key components of intervention delivery, (3) role of goal setting, (4) importance of partnership and (5) impact of intervention delivery on parents. Our findings showed that overall parents valued involvement in the programme, acknowledging benefits and challenges, as well as aspects in which they needed further support. Hospital therapists identified various education and training needs to improve their capabilities to coach parents and to collaboratively set meaningful goals. Community therapists valued the opportunity to develop their skills in intensive intervention and were keen to see evidence‐based interventions offered in the community.

**Conclusions:**

It is possible for parents, hospital therapists and community therapists to codeliver intensive upper limb intervention programmes to children with hemiplegia. However, it is important to create a flexible programme which clearly acknowledges the roles, skills and unique contributions of parents and therapists which is conducive to truly equal partnership working in appropriate settings.

**Summary**

A key messages box should be provided with each manuscript. This should include up to 5 messages on key points of practice, policy or research. This also applies to articles solicited for themed issues.

1. Partnership between parents and therapists to deliver intensive evidence‐based interventions is possible.
2. A hybrid programme of virtual and face‐to‐face sessions offers the benefits of therapist presence and increased parental involvement, making it an optimal approach for intensive intervention.
3. Therapists should be skilled in education and coaching when supporting parents in intervention delivery and formulating meaningful, collaborative goals.
4. To improve child outcomes, services would benefit from focusing on the longer term benefits of reducing disabilities by implementing targeted evidence‐based protocolised programmes.

## 1 Introduction

Family‐centred care (FCC) is considered best practice in paediatric rehabilitation (King et al. 2004). Key components of FCC are a partnership between parents and professionals, supportive respectful treatment, the sharing of information and encouragement of equal involvement (Jansen, Ketelaar, and Vermeer 2003; King et al. 1999; Rosenbaum et al. 1998). Parent delivery of an intervention increases opportunities for repetition, intensity and practice with greater integration into the home setting than can be achieved in clinic (Mahoney et al. 2006). Parent delivery has been shown to be just as effective as therapist delivery (Novak and Honan 2019). Active parental participation in therapy can also strengthen parents' emotional coping strategies and bring them greater empowerment and well‐being (Lord et al. 2018). Conversely, less successful partnerships between parents and professionals are associated with greater parental stress, disengagement and poorer parental health and well‐being (Cohen and Mosek 2019).

The International Classification of Functioning, Disability, and Health (ICF) (World Health Organisation 2001) provides a framework for thinking about health and disability applicable to everyone. The ‘F‐words’ in child neurodisability—Function, Family, Fitness, Fun, Friends and Future—align with the ICF framework and help personalise interventions by focusing on each individual's strengths and needs (Rosenbaum and Gorter 2011). Upper limb therapies, such as bimanual training modified constraint‐induced movement therapy (mCIMT) and goal directed training, are mapped to the activity (ICF) (Jackman et al. 2020) or function (F words), domains, emphasising the importance of task‐specific interventions that enhance a child's ability to perform meaningful tasks in daily life. These approaches demonstrate that motor skill acquisition involves more than practice of a physical movement (Kantak, Sullivan, and Burtner 2008) but must also include the integration of skills into everyday activities, thereby supporting FCC.

There is substantial evidence supporting the effectiveness of certain interventions for children with cerebral palsy (CP) (Jackman et al. 2021; Novak et al. 2020). For children with unilateral CP or hemiplegia, studies show that functional improvements in a child's upper limb function can be made through high intensity task‐specific training (Dong et al. 2013; Novak et al. 2013; Sakzewski, Ziviani, and Boyd 2014; Tinderholt Myrhaug et al. 2014). Approaches such as modified Constraint Induced Movement Therapy (mCIMT) and bimanual therapy require a particularly high intensity of practice of between 30 and 40 h (Jackman et al. 2020). Embedding repetition and opportunities for practice in the child's everyday environment increases the likelihood of positive outcomes for children (Jackman et al. 2020, 2021).

Several studies have explored the effect of parental directed mCIMT (Eliasson et al. 2011; Wallen et al. 2011). Some clinical trials found high dropout rates; although parents found it worthwhile, they experienced difficulties with implementation (Wallen et al. 2011). A more recent survey of therapists delivering intervention services to infants at risk of CP identified numerous challenges to collaborative delivery, including poor communication and unsuccessful family partnerships (Gmmash, Effgen, and Goldey 2020). Parent and therapist experiences of delivering intensive interventions have sometimes been reported as difficult, time consuming and tiring (Christy et al. 2010). However, successful implementation of bimanual training by parents is possible, particularly when supported by coaching from therapists and by parents' own social network (Verhaegh et al. 2022).

To contribute evidence‐based recommendations to support successful codelivery of child rehabilitation programmes by therapists and parents, it is first necessary to understand how such therapeutic partnerships function. This study adds to limited research to date which has examined partnership codelivery. To our knowledge, this qualitative study is the first to examine both parents' and therapists' perspectives on codelivering intensive upper limb interventions for children with CP. Specifically, this study (1) explores the experiences of parents and therapists when engaged in codelivering intensive upper limb intervention for their child with hemiplegia and (2) identifies areas of practice improvement to better support parent‐therapist partnerships and aid the successful codelivery of interventions.

## 2 Methods

### 2.1 Theoretical Frameworks

The study is part of a larger project using Experience‐based Codesign (EBCD), a participatory action research approach (Robert et al. 2015) that draws upon the concepts of ‘user involvement’ and ‘user experience’ for service improvement or intervention development. Semistructured interviews were selected as the most appropriate way to allow participants to share their experiences of partnering in rehabilitation delivery (Creswell and Poth 2018; Green and Thorogood 2018).

**FIGURE 1:**
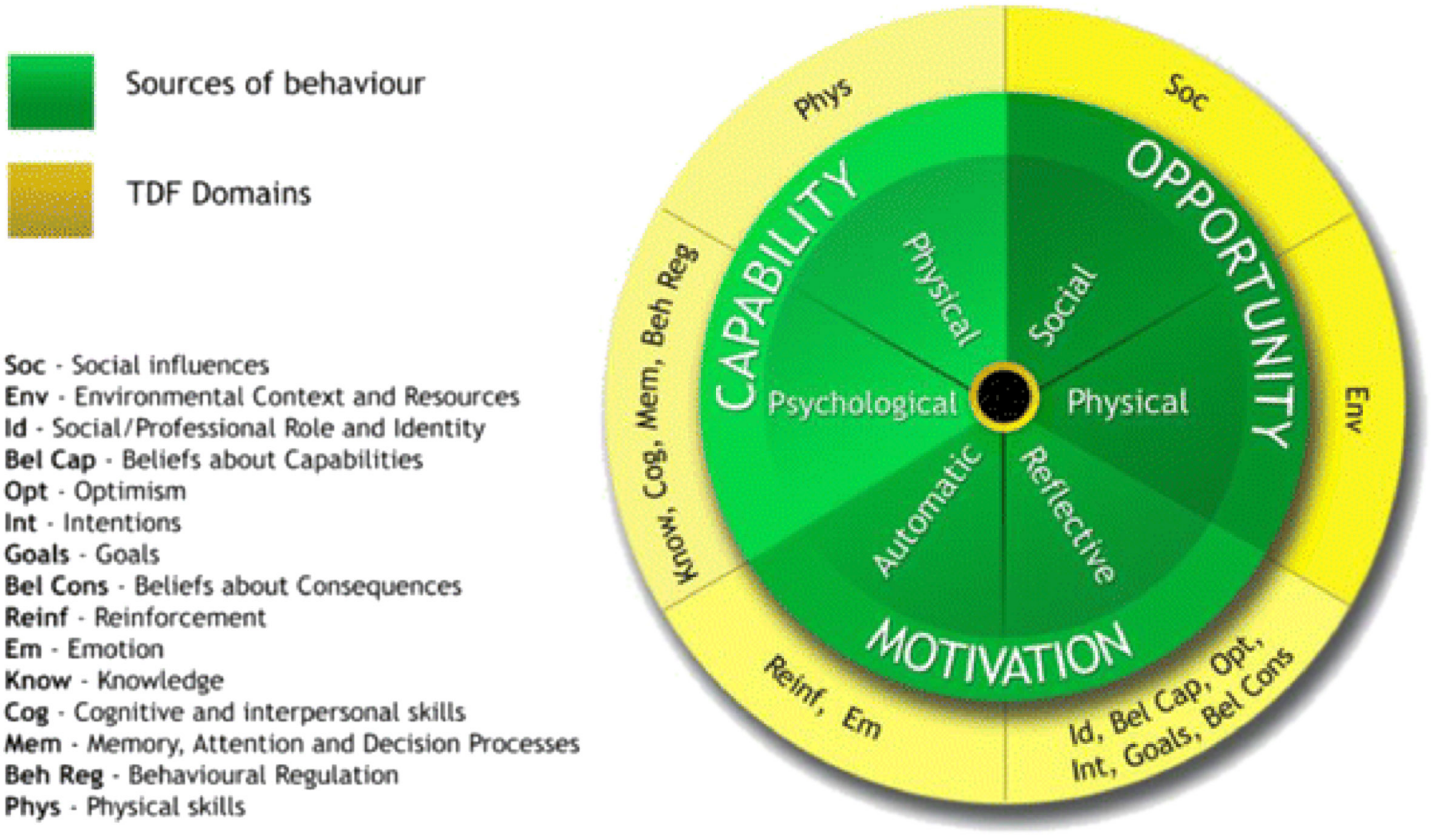
Theoretical domains framework (Atkins et al. 2017).

Previous research indicates that the successful delivery of interventions by parents is likely to require a shift in both parent and therapist behaviour (Di Rezze et al. 2013; King et al. 1999). The Theoretical Domains Framework (TDF) (Atkins et al. 2017; Michie, Atkins, and West 2014) was therefore used to map the interview data. The TDF (See Figure 1) provides a framework of constructs that are common determinants of clinical behaviours or practices (Cane, O'Connor, and Michie 2012). This ensured that any identified themes could be characterised in behavioural terms and recommendations to improve practice would be theoretically driven (Michie, Atkins, and West 2014). This paper is reported using the Standards for Reporting Qualitative Research (SRQR) (O'Brien et al. 2014).

### 2.2 Research Setting

The research was based in a National Health Service (NHS) outpatient rehabilitation service in a tertiary children's hospital in London, UK. This protocolised service embodies the elements of codelivery in partnership with parents, hospital‐based and community‐based therapists. The service delivers distributed model, evidence‐based programmes for children from 6 months to 16 years (but typically infants and pre‐school children). It includes mCIMT, bimanual training (BIT), hybrid (mCIMT and BIT) and goal directed training. These are collectively referred to as ‘intervention’ in this study.

**FIGURE 2:**
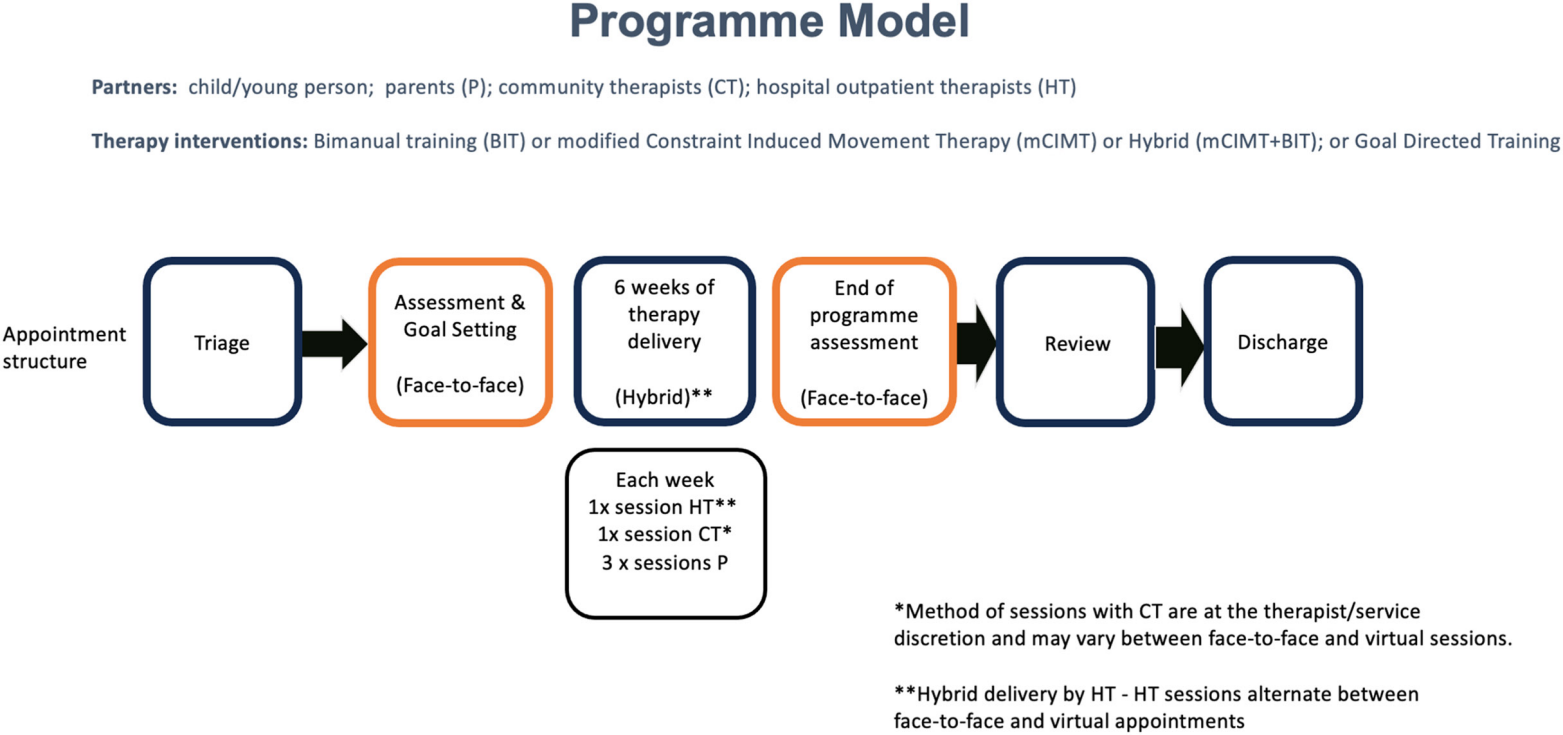
Programme model.

The programme requirements, including expectations of parent partnership in the delivery of the intervention, are verbally shared with parents when they are offered a programme. The 6‐week intervention programme includes one weekly session each with hospital‐based and community‐based therapists, plus three home sessions led by parents. Programmes are typically delivered through a combination of face‐to‐face and virtual sessions. The service moved to a combination of face‐to‐face and virtual sessions during the COVID pandemic; this hybrid approach was subsequently adopted following feedback from parents as the preferred mode of delivery (See Figure 2).

### 2.3 Ethics

Approval was obtained from the Health Research Authority (HRA) and Health and Care Research Wales (HCRW) reference 22/SW/0060 Integrated Research Application System (IRAS). Written consent to participate in the study was obtained from therapists and parents.

### 2.4 Recruitment of Participants

Seventeen families triaged by the service and offered an outpatient intervention programme between July 2022 and February 2023 were identified as eligible. This purposive convenience sampling approach aligns with the EBCD methodology, which recommends the recruitment of up to 15 patients during the ‘gathering experience’ phase of the codesign approach (The Point of Care Foundation 2001). All parents of the children undergoing the intervention programme and their partnering therapists (hospital‐based and community‐based) were invited to take part. Parents and community therapists were initially informed about the study by one of the three hospital therapists working in the service and then contacted by [lead author] to provide further information if interested. Once at least one parent had consented, their respective community‐based therapists were also invited to take part. Families were included in the study even if their community‐based therapist was not able to participate.

### 2.5 Data Collection

Data collection was conducted between August 2022 and February 2023 through semistructured interviews, either via MS teams or in person according to participant choice. Interviews lasted between 30 and 104 min (average duration 77 min). Parents and their therapists were interviewed separately. Where both parents from the same family consented, they had the option of being interviewed together or individually. A topic guide (shown in Table 1) was developed, informed by an integrative review (Massey et al. 2024) which explored the barriers and facilitators to parent delivered intervention for children with CP.

**TABLE 1:** Interview guide.

**Parents**
Tell me about your experiences of partnering with therapists in your child's rehabilitation programme?
What has helped you to deliver the programme?
What have been the main challenges you have faced?
How did your knowledge and skills in delivering the programme change from the beginning to end?
Can you tell me about your experience of setting goals for the programme?
How would you describe the relationships between all partners during the rehabilitation programme?
How has being in the programme impacted you and your day‐to‐day life?
Can you suggest ways in which partnering in the delivery of rehabilitation could be improved?
**Therapists**
Tell me about your experience of partnering with parents and therapists in the rehabilitation programme?
What are the key benefits/challenges you see for (a) you/your service and (b) parent/child?
How do you support the parents to develop their knowledge and skills?
Are there areas you as a therapist feel you need to develop when partnering in the programme?
Tell me your experience of setting goals in the programme?
Can you tell me about the relationship between you; the community/Evelina therapists; parent and child when partnering in the programme?
Can you suggest ways in which the partnership delivery of rehabilitation could be improved?

### 2.6 Trustworthiness of the Research

To ensure the trustworthiness of the research, we adhered to specific criteria to ensure credibility, dependability, confirmability and transferability (Lincoln and Guba 1985; Nowell et al. 2017). All transcripts were sent to participants for member checking (Birt et al. 2016). To promote credibility, the topic guide was piloted with a parent and a therapist representative. Records of data were collected, alongside field notes and transcripts to ensure an audit trail supporting dependability (Nowell et al. 2017). The lead author kept a reflexive journal throughout and had ongoing discussions with co‐authors to establish confirmability. Having previously worked in the service as an occupational therapist, they reflected on how this prior experience might influence her perspectives on parent and therapist partnership. They were mindful of the potential impact on the approach to conducting interviews and analysis. To minimise bias, external perspectives were sought from experienced qualitative researchers in sociology and anthropology (G.R. and V.T.) with no affiliation to the service. This commitment to reflexivity enhances the trustworthiness of the data (Lincoln and Guba 1985).

### 2.7 Data Analysis

Data analysis was initially completed separately for parent and therapist interviews. Reflexive Thematic Analysis (RTA) (Braun et al. 2022) was used due to its flexibility and acknowledgement of the active engagement of the researcher in knowledge production (Braun et al. 2022). Data familiarisation began by reading and re‐reading the transcripts. Initial inductive coding by the lead author was completed using NVIVO 14.23 (QSR International Pty Ltd 2018; Lumivero 2023). Coding schemes were reviewed and discussed with co‐authors V.T. and G.R. Finalised codes were exported to MS Word for theme development and revision. Team discussions involving J.M., V.T. and G.R. played a pivotal role in facilitating the interpretation of emerging themes. The iterative process of theme refinement and definition was continued until consensus was reached by co‐authors.

In the final phase, themes from the therapists and parental dataset were compared to determine similarities, differences and relationships between the data. The triangulation of data sources during analysis enabled validation of identified themes (Nowell et al. 2017). The subsequent mapping of the themes to the TDF (Michie, Atkins, and West 2014) allowed the development of an initial set of behavioural recommendations for practice and to help inform future interventions to foster parental and therapist partnerships.

## 3 Results

Ten of 17 families approached took part in the study; in five cases, the entire team of parents and therapists (community and hospital) were recruited. In all other cases, community therapists could not be recruited because of ethical approval delays at sites or slow responses to study invitations. In total, twelve parents (eight mothers and four fathers—two parent dyads) and eight occupational therapists (three hospital‐based and five community‐based) were interviewed. Children were age 16 months to 4 years. Children's perspectives were not included given their age; the focus was on parents/carers as key partners in this study. Of the seven families not participating, six had time constraints, and one mother, nearing the end of her second pregnancy, preferred not to participate. Participant characteristics are shown in Table 2.

**TABLE 2:** Participant characteristics.

Study identifier	Child Age	Child gender	Birth order	Childs diagnosis	Employment status	Parent interviewed	Therapist interviewed
1 (002)	18 months	Female	2	Hemiplegia	Part time employed	Mother	H & CT
2 (003)	18 months	Female	1	Hemiplegia	Full time employed	Father	H & CT
2 (003)					Maternity leave	Mother	H & CT
3 (004)	3 years	Female	1	Hemiplegia	Full time	Father	H
4 (005)	4 years	Female	2	Four limb cerebral palsy	Full time	Mother	H & CT
5 (006)	3 years	Male	1	Hemiplegia	Not employed	Mother	H & CT
6 (010)	2 years	Male	4	Hemiplegia	Full time employed	Father	H
7 (013)	3 years	Male	2	Hemiplegia	Full time employed	Mother	H
8 (014)	16 months	Male	1	Hemiplegia	Part time employed	Mother	H & CT
					Full time employed	Father	
9 (016)	4 years	Female	2	Hemiplegia	Not employed	Mother	H
10 (017)	20 months	Female	1	Evolving unilateral motor disorder	Not employed	Mother	H

Therapists			
Study identifier	Gender	Experience in current role	Caseload^a^
HT‐1	Female	1 year	Specialist caseload: Hemiplegia, childhood stroke
HT‐2	Female	2 years	Specialist caseload: Hemiplegia, skeletal dysplasias
HT‐3	Female	2 years	Specialist caseload: Hemiplegia, neonatal stroke
CT‐1	Female	4 years	Mixed caseload (early years complex needs 0–3 years, mainstream schools supporting Education Health Care Plans (EHCP)
CT‐2	Female	5 years	Mixed caseload (complex physical disability under 5 years, delay,
CT‐3	Female	6 years	Mixed caseload (complex physical disabilities, hemiplegia, neurodevelopmental disorders, genetic conditions, autistic spectrum disorder, developmental delay)
CT‐4	Female	11 years	Mixed caseload (complex physical disabilities, hemiplegia neurodevelopmental disorders, autistic spectrum disorder, developmental delay)
CT‐5	Female	4 years	Mixed caseload

^a^ Typically hospital‐based therapists have a specific caseload whereas community therapist caseloads involve children with a range of different needs and in varying settings.

Five major themes were identified across the parent and therapist datasets: (1) realities of accessing intensive intervention, (2) key components of intervention delivery, (3) goal setting, (4) importance of partnership and (5) impact of intervention delivery on parents. Illustrative quotes from parents, community therapists and hospital therapists are presented in Table 3. In the following sections, we present the main findings and highlight the important related subthemes in turn.

**TABLE 3:** Illustrative parent and therapist interview quotes.

Theme 1. Realities of accessing intensive intervention		
Subthemes	TDF	Quotes
1.1 Availability of services	Environmental context and resources	*‘The local therapists, they did not really offer anything beyond just sort of general advice like, you know, encouraging him to use that hand, which they do they offer other things, you know, they offer, like, access to equipment’ (P5)* *‘a lot of parents, they are quite frustrated because they have not had a lot of community input’ (HT2)*
1.2 Time and financial resources	Environmental context and resources	*‘the downsides to it, are, the travel, the cost of travelling …*. I*'m having to take pretty much a day off work to go into London’ (P3)* *‘I think there's a lot of commitment and a lot of juggling for them, if they have other children and work commitments’ (CT1)*

Theme 2. Key components of intervention delivery		
2.1 Parents' experiences of learning	Knowledge, skills	*‘The therapist at the hospital were very good at showing [my child] first and that's what they taught us to do. So, showing him and explaining and using the right language with him’ (P7)* *‘we had a sort of kick off session … Then it's sort of off you go … I feel like we are kind of left to our own devices in a fairly big way’ (P2)* *‘a lot of trial and error to see which toys they are [the child] motivated by … .sometimes within our sessions, mum or dad would come sit alongside me and would both be engaging with this little guy’ (CT5)*
2.2. Involving and supporting parents	Knowledge, Skills	‘*I think it was always just understood that we go in and I kind of because of how the room is set up and I am sat to the side …. I have to be honest and probably took the opportunity just to let them just do it and just have a bit of a break’ (P5)* *‘I love that I get to do therapy, because we do not always get to do it. Half the time, I feel like I'm assessing and giving fly by night information that does not always get heard, but because I'm now given the opportunity to provide regular input and do therapy, I get the joy of being a therapist again’ (CT3)* *‘I think there's always skills to learn around my knowledge, evidence base behind bimanual programmes or constraint programmes and motor learning theory and all the evidence that's emerging around that, also around how to support and engage with families in the best way possible and what they need or benefit the most from and how I can tailor my intervention to support that’ (HT1)*
2.3 Sustaining the child's engagement	Skills	*‘..keeping her interested, so that's a challenge … we have made it fun for her as much as possible, and she's always laughing in the session. So, um, I think the challenge is just to keep that up’ (P3)* *‘their ability to engage with their child and play with their child. I think that can really vary. Some parents get it like that so quickly and really take on board the strategies and come up with great ideas and other parents I think find it a bit more difficult to, just to play with their children’ (HT3)* ‘*can I really keep a two year old there [sitting at a table] that long? …*. *it was a real eye opener to me that actually you can’ (CT4)*
2.4 Peer and family support	Social influences	*‘I'm fairly certain that that has helped with regards to, like that motivation and getting fed up or bored or tired. I think the fact that, um, you know, we can consistently both be there. So if one of us is having a bad day, um, not feeling too great, Um, then the other one can kind of keep the fun going’ (P3)* *‘[Hospital] therapist has been brilliant when our daughter [other child] was at home the other day, she engaged her in the session. Which is brilliant because I know our daughter does not feel like she's, she's left out’ (P7)* *‘family support and involvement is quite important’ (CT5)*

Theme 3. Role of goal setting		
Subthemes	TDF	Quotes
3.1 Realistic goal setting	Knowledge, Skills	*‘it's tricky to know what the right level that you should be aiming for is, I just did not know what to suggest … I just felt a bit like, well, I should know what we want to achieve … what he is able to achieve with the right support, but I did not really. I ended up relying on them’ (P5)* *‘it's difficult to really drill down to what we actually are working on here and saying it in, like, a simple way that makes sense to parents and relating it to a functional goal that's realistic’ (CT1)* *‘with some parents it can be really, really difficult for them to think of goals … I do not want to set the goals for them. So it's trying to help guide them without putting the goal in their mouth’ (HT3)*
3.2 Managing expectations	Belief about consequences	*‘I think naturally, as a parent, you think that something is going to be a golden bullet with all of this stuff … You know, little things like that are huge gains. And I think you underestimate or you undervalue things like that in the beginning because you just, yeah, it takes a while to kind of get your head round’ (P7)* *‘… expectations is a huge one that parents bring with them, the expectations that they have for their child, expectations that they have for themselves in delivering the programme’ (HT1)*

Theme 4. Importance of partnership		
4.1 Collaboration between services	Skills	*‘[I] do not know what the local therapists are really party too. In terms of, do they have a call with the team? How in depth is that call? Do they get updates on what's being done, what's been achieved…? I do not know what part they play ‐‐ apart from me setting a session and them doing their normal day to day thing to provide that session’ (P10)* *‘I need to remember to always send the communication. I'm bad at that.. I fill in the logbook because the family will always bring in. So I fill that in. It's just the sort of liaison back to the OT. I start off quite well, and then the weeks go by and I'm like Oh I have not done it yet’ (CT3)* *‘sometimes because of [community therapist] work schedules, sometimes we do not get updates in a timely manner … ..it poses the challenge that I'm not sure how the child is progressing in their sessions..’ (HT1)*
4.2 Value of strong connections	Social influence	*‘the therapists are just amazing with [my child]. Just, so enthusiastic. It's the enthusiasm, the way they talk to him, like it's really natural … genuine interest in [my child] which has been lovely’ (P7)* *‘I think maybe sometimes to build that relationship between the community OT and the parent because sometimes they might not have that much input in the community’ (HT3)* *‘as community therapist … I can also carry that back into the nursery or into other activities of daily living where I can also then guide the family. So I've maybe got more influence in those more extended activities’ (CT5)*
4.3 Sharing expertise	Professional/social role and identity	*‘we are the only ones that can calm her down, they are not going to be able to give her a hug. They're not the ones that can call her a silly name or do something silly and make her giggle, or they can to some extent, but actually, sometimes she's only going to be reassured by one of us or seeing her sister or something and actually, yeah, we are the ones who are familiar with her, and I think that actually is quite important’ (P2)* *‘Together, [this] programme brings a real speciality and expertise that can really guide me as a community therapist, because I think although this is an area of my job and it's an area that we have been trained in, and there's definitely lots to read about, it forms a small section of my job, and I think just really bringing in that focused expertise for them [hospital therapists]’ (CT5)*

Theme 5. Impact of intervention delivery on parents		
5.1 A confronting experience	Emotion	*‘it just sort of makes you flash forward like 18 years. And, you know, is she ever going to get married? Is she ever going ‐‐ you know, it's all of that stuff and I find that quite difficult, … She's a disabled, differently abled child with additional needs. Her life, whether extreme or not, is not going to be the same as her sister's. She does not have the same opportunities’ (P2)* *‘a thought I had so many times during it was, if he was normal, ‘normal’. If he did not have this, what would we be doing now? There are so many other things we could be doing now that would be more fun than this. And it's not fair that he has to do this. Life is not fair’ (P5)* *‘the mum was quite emotional throughout the six weeks, because, you know, it's really focusing, is not it in on the difficulties that that child is having?’ (CT1)*
5.2 Gaining confidence and being empowered	Belief about capability	*‘it's really nice to be able to help her and actually like … moments of like pride where you feel proud of her for doing it. But you are also proud of yourself that you are able to help her’ (P3)* *‘it's really nice to see when parents kind of have the confidence to come in and say, Well, look, I can show you what I can do with them or what they have been doing with me’ (HT3)* *‘… because of my confidence with it because I've been with the [hospital therapist]. I had a conversation with our Therapy Skills manager who was saying, well, we cannot do intensive weekly for six weeks, and I said, Well yes, but if we did this for six weeks, we might not see them again for another six months. So, we are doing all those monthly appointments in one blitz and go, and I do not know that I would have had the confidence to say that if I had not done it … We're going to write a new, a specific pathway for intensive intervention, with constraint and bimanual skills’ (CT4)*

**Theme 1: Realities of Accessing Intensive Intervention**

***TDF: Environmental Contexts and Resources***

**1.1 Availability of Services**

All parents appeared to identify challenges in accessing therapy services with their children, identifying long waiting times for community therapy services. Several reported delays in gaining a diagnosis for their child. Once community services were in place, the provision was described as lacking intensity with infrequent appointments. Some parents resorted to private sector care, and hospital therapists were aware of parental frustration with slow or inadequate community services provision.

**1.2 Time and Financial Resources**

All parents reported that travelling to face‐to‐face intervention sessions cost them time, financial resources and work disruptions. Parents and therapists also acknowledged the challenges associated with fitting home sessions around the needs of work, family and school. Parents appreciated it when the scheduling of the 6‐week program considered the needs of both the child and the family. In‐person appointments for infants often meant parents needed to adapt their infant's routine or find a time when their child was most receptive.

**Theme 2: Key Components of Intervention Delivery**

***TDF: Knowledge, Skills and Social Influence***

**2.1 Parents' Experiences of Learning**

Parents seemed to find that partnership working had increased their skills in delivering the intervention and confidence in implementing strategies beyond the end of the programme. Collaboration with therapists appeared to provide them with greater insights into their child's capabilities within therapy and in daily life. Most parents reflected that monthly or bimonthly reviews would be beneficial to monitor progress, reset goals and refresh activity ideas to progress their child's upper limb function and sustain their engagement.

**2.2 Involving and Supporting Parents**

Several therapists spoke of modelling (exhibiting specific behaviours, feedback, skills) and demonstration of the therapeutic activities to show parents, although some reflected on how they could improve their skills in coaching, facilitating parental involvement and communicating evidence. It appears that all participants consistently noted that face‐to‐face sessions were predominantly therapist‐led, with parents primarily observing. Some parents understandably used therapist observation as a break from interacting directly with their child. The clinic environment did not always lend itself to enabling parents to lead or codeliver, even in terms of the way that seating and resources were set up. Interestingly, virtual sessions shifted the dynamic and made it easier for the parent to lead the session with therapist feedback. Some parents felt that virtual appointments were less effective as their child was easily distracted or less engaged than with the therapist. Others valued the hybrid programme delivery which offered them greater flexibility than all clinic‐based sessions.

**2.3 Sustaining the Child's Engagement**

It seems that both parents and therapists identified challenges in maintaining children's engagement throughout the intervention. Parents found it challenging to determine the appropriate level of encouragement, particularly when their child was less engaged. Therapists reported that parents commonly sought advice on maintaining their child's engagement, which often depended on the parents' comfort in embracing their own playful side. Most parents were able to learn techniques from therapists while bringing their own knowledge of the child to make sessions more entertaining.

**2.4. Peer and Family Support**

Parents gave the impression that having both parents and sometimes grandparents involved in delivery helped with motivation and energy and helped share the programme load. Parents appreciated therapists who actively engaged siblings in sessions which added novelty to the intervention and eased parents' concerns that other children were being left out. Parents also wanted the opportunity to share their experiences and strategies with other parents who were codelivering interventions. Therapists agreed that parents may benefit from this type of peer support and that it would be a good way of sharing practice.

**Theme 3: Role of Goal Setting**

***TDF: Skills, Beliefs About Consequences, Belief About Capabilities***

**3.1 Realistic Goal Setting**

Parents and therapists both highlighted the challenge of determining realistic, feasible programme goals. Some parents felt that goal setting was the therapist's responsibility, whereas others wanted to be more involved in goal formulation. Therapists acknowledged that parents' ability to set goals was variable, and it was sometimes difficult to guide them towards formulating realistic targets. Not all parents were clear about the importance of setting goals. For example, one parent suspected that the goals existed to inform the clinical service rather than for the child's benefit. However, the evident progress in the child's arm and hand function served as a continual source of motivation for parents and therapists to continue.

**3.2 Managing Expectations**

It could be inferred that realistic goal setting is heavily dependent on understanding what progress can be achieved given a child's functional limitations. Several parents expressed difficulty in knowing what outcomes to expect from the intervention. Therapists recognised that parents often had unrealistic expectations of themselves and of their child on commencing the programme, and it was sometimes difficult to manage these without discouraging their enthusiasm.

**Theme 4. Importance of Partnership**

***TDF: Skills, Knowledge and Social Influence***

**4.1 Collaboration Between Services**

The programme required the collaboration of hospital‐based therapists, community‐based therapists, and families. Parents seemed to have a clearer understanding of the role of the hospital therapists, as specialists, compared to the contribution of community therapists. All partners reported communication challenges, particularly with regard to progress reports between the community‐based and hospital‐based therapists. Parents were unclear of the communication channels and observed a general lack of joined‐up working between services.

**4.2 Value of Strong Connections**

Parents and therapists appeared to agree that child/therapist rapport was an essential component of intervention delivery. Seemingly, a strong relationship between parents and therapists allowed them to feel comfortable to ask questions and share ideas between sessions. Parents reported generally positive relationships with their hospital therapists. Community therapists appreciated the regular weekly contact with families which provided further opportunities to consolidate their relationship. However, parental expectations of the community therapists in the intervention were clearly affected by prior experiences of inadequate community provision of OT services. Hospital therapists recognised this issue and the importance of supporting a closer relationship between community therapists and parents to optimise the intervention.

**4.3 Sharing Expertise**

All partners were able to identify the expertise that others brought to the partnership model. Therapists in particular identified parents as experts on their child, knowing their routines, predicting their needs and identifying what motivates their child. All therapists reported gaining new and creative ideas from parents and other therapists. Most community therapists enjoyed learning specialist skills from the hospital team and were aware of their opportunity to embed the intervention into nursery and school life beyond the partnership programme.

**Theme 5: Impact of Intervention Delivery on Parents**

***TDF: Emotion, Belief About Capabilities***

**5.1 A Confronting Experience**

Parents reported that witnessing their child's limitations in intervention sessions made them concerned about what the future might hold. This included whether their child would be bullied or have limited opportunities because of their hemiplegia. Some parents seemed resentful that intensive intervention at such a young age had disrupted their time to just enjoy their baby. Therapists confirmed the strong emotional impact that watching their child struggle during therapy could have on parents. However, they also recognised parents' commitment and determination to delivering their child's therapy effectively.

**5.2 Gaining Confidence and Being Empowered**

It seems that while some parents continued not to feel like ‘experts’ in intervention delivery, for others, there was a clear sense of empowerment from codelivering their child's programme. It is possible that several mothers felt the bond with their child had deepened through dedicating time to deliver intervention. There is an indication that several community therapists reported greater confidence after partnership working with hospital therapists. It can be noted that some became passionate about advocating for changes within their local service to support similar types of intensive programmes.

**FIGURE 3:**
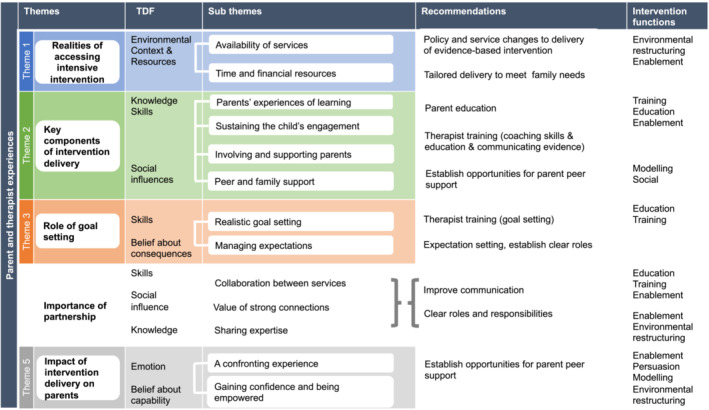
Themes mapped to TDF with recommendations for practice.

The 14 subthemes outlined above were mapped to eight of the fourteen domains of the TDF, and the resulting practice recommendations are presented in Figure 3.

## 4 Discussion

This study aimed to examine parents' and therapists' experiences when partnering in the delivering intensive upper limb interventions and to provide practice recommendations. Codelivered programmes involving parents and therapists offer greater opportunities for repetition and practice in real‐life settings. They also support the principles of FCC (Jansen, Ketelaar, and Vermeer 2003; King et al. 1999; Rosenbaum et al. 1998). Previous studies have shown variation in parents' capacity to implement intervention in daily life (Smidt, Klevberg, and Oftedal 2020; Chamudot et al. 2018; Peplow and Carpenter 2013). This study did not measure adherence to intervention fidelity or intensity. However, the findings suggest that parents were willing to lead sessions despite practical challenges.

Consistent with previous research, parents faced challenges like finding practice time, travelling to appointments and managing the financial impact of taking time off work. (Beckers et al. 2021; Kruijsen‐Terpstra et al. 2016; Ross and Thomson 1993). Given these circumstances, service flexibility was highly valued, with many parents preferring virtual sessions for this reason. However, some parents felt that children were less engaged without a therapist's presence. Our study found that virtual sessions required parents to take the lead in delivering the intervention. A hybrid approach, combining virtual and in‐person methods, may offer an optimal solution for both parents and therapists.

Managing expectations and setting appropriate goals were reported as challenging by both parents and therapists, a difficulty also highlighted in previous research. (King, Wright, and Russell 2011; Playford et al. 2009; Rosewilliam, Roskell, and Pandyan 2011) It is possible that a few parents felt that intervention goals did not capture their child's progress. They also found it difficult to anticipate likely outcomes, possibly due to lack of knowledge of their child's expected trajectory. This suggests an opportunity to educate parents. This aligns with research showing that parents, especially of young children, often feel uncomfortable setting reasonable and attainable goals due to their limited knowledge of their child's condition and relevant intervention approaches (Wiart et al. 2010). It is evident that goal setting is important as it is the foundation for evidence‐based therapy and the initial stage in rehabilitation. The Evidence bAsed Decision Making tool (READ model) outlines a clinical reasoning framework beginning with parent‐led goal setting and can support therapist decision making (Novak et al. 2021). Tools such as the Canadian Occupational Performance Measure (COPM) and Goal Attainment Scaling (GAS) (King et al. 2000; Bovend'Eerdt, Botell, and Wade 2009) can support goal setting.

It seems that parents felt they learned skills by watching the therapist and commonly reflected the clinic environment was not conducive to them leading or participating in intervention delivery. Our study suggests that therapists still took on the lead role during face‐to‐face sessions, which is akin to a more traditional philosophy on intervention delivery (Smith and Samuels 2021). Current philosophies emphasise coaching parents to foster their involvement as active and equal partners in the intervention process, empowering families to make informed decisions (Akhbari Ziegler, Dirks, and Hadders‐Algra 2019). Coaching has been used in parent‐implemented interventions (Friedman, Woods, and Salisbury 2012). Recent studies underline the application of coaching to support families with infants or young children in early intervention (Morgan, Badawi, and Novak 2023; Akhbari Ziegler, Dirks, and Hadders‐Algra 2019). However, recent reviews have identified ambiguities in coaching definitions, the distinction between education and coaching and inconsistent terminology, which hinders the integration of coaching into practice (Schwellnus, King and Thompson 2015; Ward et al. 2019; Kemp and Turnbull 2014; Akhbari Ziegler and Hadders‐Algra 2020).

Our findings suggest that therapists did not always use coaching principles or consider how the learning environment could facilitate parents to codeliver. It might be that therapists could benefit from further training in evidence‐based educational, coaching and motivational techniques to boost parents' confidence and foster their involvement (Reeder and Morris 2016). It is possible that developing parent knowledge through education and therapeutic skills is essential for supporting them in delivering interventions (Massey et al. 2024). A combination of education, coaching and skill development may be helpful in supporting parents in strategies such as optimising their child's attention during the intervention and implementing behaviour strategies, as highlighted in this study.

There is a suggestion that a greater understanding of how to meet individualised learning preferences would enhance the intervention resources provided to parents (Hurtubise and Carpenter 2017). Our study also supports previous research suggesting that while parents enjoy maximising their child's potential (Kruijsen‐Terpstra et al. 2013) they may also experience difficult emotions when confronted with their child's limitations (Harniess et al. 2023). Thus, therapists need to consider parents' emotional responses to codelivery when planning interventions. Providing opportunities for parents to support each other may be beneficial in this respect and could be facilitated by therapists (Piggot, Paterson, and Hocking 2002). Equally, occupational therapists' dual training in physical and mental health should equip them to consider the psychological needs of families.

It seems that despite the collaborative ethos of the service, the partnership components of equal voice, communication, mutual learning and acknowledgement of partners' expertise were not always evident in our participant data (Joseph‐Williams, Edwards, and Elwyn 2014). Our results suggest that there was continued deference by parents and community therapists to the hospital team as ‘knowledge experts.' It is possible that parental frustration with the prior poor provision of community services affected their perspectives of community therapists joining the programme. It appears that most community therapists reported increased confidence at the end of the programme. They also recognised an important role in further supporting parents' practice at home, school, nursery and beyond the formalised programme (Morgan et al. 2016). However, all partners reported communication challenges, which hindered elements of partnership working. It seems apparent that providing clearer role definition and expectation management for all parties at the beginning of the programme, with an emphasis on each one's unique skillset, may encourage movement towards equal partnership.

Given the importance of maximising neuroplasticity through access to best‐available evidenced intervention (Morgan et al. 2016; Jackman et al. 2021; Novak et al. 2017), reported delays in diagnosis and access to evidence‐based intervention is concerning. It is possible that access issues are driven by staff recruitment and retention and commissioning priorities, which are internationally recognised challenges (Feldman et al. 2002; Royal College of Occupational Therapists 2022). While evidence‐based interventions may be offered in some community services, we suggest that they may not replicate the protocolised programme valued by parents and therapists in this study. Our findings suggest that community therapists were prompted by the positive clinical outcomes of these interventions to advocate for similar programmes within the community. Parents also appreciated the potential benefits for upskilling community therapists to deliver intensive upper limb intervention. Such an approach could enable greater access for children and families in alignment with policy objectives (Alderwick and Dixon 2019) across a range of disabilities/diagnoses. Commissioning models, which focused on the long‐term benefits of reducing disabilities, may foster greater delivery of targeted evidence‐based protocolised programmes.

### 4.1 Limitations

Given the limited diversity of our sample, future research involving participants from a range of backgrounds, cultures and ethnicities is necessary to understand how to tailor support to different groups. Although the lead author worked historically in the participating hospital service, a reflexive, team approach was adopted to analysis to minimise potential bias. However, hospital therapists might have been more likely to agree to participate and it might have affected what they disclosed. In addition, while using a behavioural theory to analyse the data was helpful for developing recommendations, it was sometimes challenging to map data on to a single domain of the TDF when it included multiple concepts.

## 5 Conclusion

Our recommendations for fostering successful partnership working when delivering intensive upper limb rehabilitation programmes with a child include the following: providing a flexible service and setting which encourages active parental involvement, ensuring therapists are skilled to educate and coach parents to maximise their confidence and set clinically appropriate but meaningful intervention goals, providing emotional support to parents and utilising the positive benefits of parental peer support and establishing the roles, contributions and communication requirements of different partners from the outset.

## Author Contributions

**Jill Massey:** conceptualization, investigation, funding acquisition, writing – original draft, methodology, project administration, formal analysis, writing – review and editing, resources. **Vicki Tsianakas:** writing – review and editing, methodology, supervision, conceptualization, formal analysis. **Anne Gordon:** writing – review and editing, conceptualization, supervision. **Glenn Robert:** conceptualization, methodology, validation, writing – review and editing, supervision, formal analysis.

## Ethics Statement

The study was approved by the Health Research Authority (HRA) and Health and Care Research Wales (HCRW) reference 22/SW/0060 IRAS project ID 305046.

## Consent

Informed consent was obtained from all subjects involved in the study.

## Conflicts of Interest

The authors declare no conflicts of interest.

## Permission to Reproduce Material From Other Sources

Permission for unrestricted use provided appropriate credit is given to the original authors. This is documented in the original article. Regarding Figure 1, the TDF—This is an open access article distributed under the terms of the Creative Commons CC BY license, which permits unrestricted use, distribution, and reproduction in any medium, provided the original work is properly cited. You are not required to obtain permission to reuse this article. CC0 applies for supplementary material related to this article and attribution is not required (https://s100.copyright.com/AppDispatchServlet?title=A%20guide%20to%20using%20the%20Theoretical%20Domains%20Framework%20of%20behaviour%20change%20to%20investigate%20implementation%20problems&author=Lou%20Atkins%20et%20al&contentID=10.1186%2Fs13012‐017‐0605‐9©right=The%20Author%28s%29.&publication=1748‐5908&publicationDate=2017‐06‐21&publisherName=SpringerNature&orderBeanReset=true&oa=CC%20BY%20%2B%20CC0).

## Data Availability

The data that support the findings of this study are available from the corresponding author upon reasonable request.
